# Health of Black and LGBTQIA+ Populations in Health EDUCATION: A Scoping Review Protocol

**DOI:** 10.3390/nursrep15060217

**Published:** 2025-06-13

**Authors:** Bruno Pereira da Silva, Patrícia de Carvalho Nagliate, Gabriel da Silva Brito, Danilo Bonfim de Queiroz, Ana Paula de Morais e Oliveira, Célia Alves Rozendo, Danielly Santos dos Anjos Cardoso, Roberto Ariel Abeldaño Zuñiga, Paula Cristina Pereira da Costa, Maria Giovana Borges Saidel, Eduardo Sodre de Souza, Débora de Souza Santos

**Affiliations:** 1 Multidisciplinary Center, Federal University of Acre (UFAC), Cruzeiro do Sul 69890-000, AC, Brazil; bruno.silva@ufac.br; 2 School of Nursing, State University of Campinas (UNICAMP), Campinas 13000-000, SP, Brazil; paulapc@unicamp.br (P.C.P.d.C.); mgsaidel@unicamp.br (M.G.B.S.); edusodre@unicamp.br (E.S.d.S.); deborass@unicamp.br (D.d.S.S.); 3 School of Philosophy, Languages and Literature, and Human Sciences, Federal University of São Paulo (UNIFESP), Guarulhos 02141-000, SP, Brazil; g.brito@unifesp.br (G.d.S.B.); danilo.bonfim@unifesp.br (D.B.d.Q.); 4 School of Nursing, Federal University of Alagoas (UFAL), Maceió 57000-000, AL, Brazil; patricia.nagliate@eenf.ufal.br (P.d.C.N.); celia.rozendo@gmail.com (C.A.R.); danielly.anjos@eenf.ufal.br (D.S.d.A.C.); 5 Library of the Faculty of Medical Sciences, State University of Campinas (UNICAMP), R. Albert Sabin, s/ nº. Cidade Universitária Zeferino Vaz., Campinas 13083-894, SP, Brazil; arvigui@unicamp.br; 6 Postgraduate Department, University of Sierra Sur (UNSIS), Miahuatlán de Porfirio Diaz, Oaxaca 70805, Mexico; 7 Helsinki Institute of Urban and Regional Studies, University of Helsinki, 00150 Helsinki, Finland; 8 Centre for Social Data Science, Faculty of Social Sciences, University of Helsinki, 00150 Helsinki, Finland

**Keywords:** continuing education, professional training, teaching, black people, sexual and gender minorities, health inequalities, scoping review

## Abstract

**Introduction:** The health education curricula should explicitly recognize, define, and address the unique needs and health disparities faced by Black and LGBTQIA+ populations, as a means of ensuring that healthcare for these populations is both comprehensive and inclusive. **Aim:** To map scientific evidence and identify knowledge gaps regarding the health of Black and LGBTQIA+ populations within the global context of health education. **Methods:** A scoping review will be conducted following the JBI methodology. The articles will be retrieved from Scopus, Web of Science, PubMed, Embase, MEDLINE, BVS, CINAHL, ERIC, Cochrane, BDTD, PQDT, EBSCO, and NDLTD. The search will be conducted without language or time restrictions. Two independent reviewers will screen the studies and extract data using a form specifically developed for this purpose. The concepts, definitions, structures, results, and applications of professional health education worldwide for the healthcare of Black and LGBTQIA+ populations will be summarized and discussed. **Inclusion Criteria:** Studies related to professional health training at both undergraduate and graduate levels, as well as other educational modalities that address the provision of healthcare for these populations, will be included. The results will be presented in both tabular and graphical formats, accompanied by a narrative summary. Protocol registered in the Open Science Framework (OSF).

## 1. Introduction

The vulnerability of Black and/or LGBTQIA+ people places them among the groups with the worst health outcomes. Such vulnerability is explained by the structural inequalities that restrict access to resources and protection strategies, which are related to the social determination of the health-disease process [1].

Health training, whose gaps reflect and reproduce these inequalities, can be analyzed from an intersectional perspective, especially with regard to the neglect of these issues in the curriculum. This draws attention to the experiences of illness and avoidable deaths of Black and LGBTQIA+ people as a result of these inequalities.

In this sense, adopting intersectionality as an analytical framework allows us to broaden our exclusively biomedical perspective to include the structural aspects that determine these unequal experiences.

With attention to this point, intersectionality will be adopted in this review as an analytical resource that is based on Critical Social Theory [2]. On the one hand, this review also seeks to deepen the understanding of unique and collective experiences that result in oppression and privilege, operated by combined logics of social markers of differences such as race, gender, sexuality, class, and others. On the other hand, it problematizes its “political praxis” dimension as a resource for mobilizing strategies to transform unequal health scenarios through recommendations for formulating public policies in health training that tackle structural and institutional inequalities, promoting health equity for historically marginalized populations [3].

The “complex knot” that determines different social places for different people and groups, such as Black or LGBTQIA+ people, in the context of health training, reflects deficiencies that are ultimately the result of exclusionary logics and that impact their health conditions and access [2].

In the case of the Black population, racism, manifested institutionally, historically and culturally, affects interpersonal relationships and systematically places this group at countless disadvantages in all dimensions of individual and collective life. In health, for example, its effects are devastating, perpetuating exclusion, neglect and institutional violence, expressed by morbidity and mortality indicators [4,5,6].

With regard to the LGBTQIA+ population, in the face of the specific needs of this group, barriers are imposed that are determined by systematic forms of discrimination and social exclusion, which can be translated as institutionalized LGBTQIA+ phobia. This poses challenges that directly impact health promotion and disease prevention [6].

Health training focused on vulnerability care, as proposed by Buss et al. [1], is fundamental for historically marginalized populations, such as Black and LGBTQIA+. However, this approach is often neglected in undergraduate health courses, compromising the training of health professionals to deal with such complexities [7,8]. The inclusion of SPN and LGBTQIA+ in health curricula is still limited. When it is present, it occurs as a sub-theme without a specific or mandatory approach [9,10]. This scenario compromises the training of health workers and their ability to provide adequate and humanized care that meets the needs of these populations [8,11].

It is essential that nation-states and international organizations, such as the UN, UNESCO, PAHO, and WHO, expand the recognition of ethnic-racial and gender-based violence, promoting the integral health of Black and LGBTQIA+ populations. Although initiatives already exist, policies need to be strengthened to increase their impact and effectiveness.

International empirical evidence reinforces the health inequalities experienced systematically by Black and LGBTQIA+ populations in various global contexts. According to the World Health Organization (WHO), structural racism and discrimination based on sexual orientation and gender identity are social determinants of health that directly contribute to barriers to accessing health services, higher prevalence of communicable and non-communicable diseases, mental health problems and lower life expectancy among these groups [12]. The Pan American Health Organization (PAHO) also documents significant inequalities between people of African descent and LGBTQIA+ individuals in the Americas, including high rates of maternal mortality, HIV infection and mental distress, often exacerbated by institutional stigma and the absence of culturally competent care [13]. This data reinforces the urgency of incorporating intersectional approaches into health training that simultaneously take into account the ethnic-racial and sexual and gender diversity dimensions, in line with the United Nations 2030 Agenda, which establishes the reduction in health inequities as a global priority.

Health practice must be based on a political–ideological commitment to valuing life, combining technical–scientific knowledge, competence, and cultural sensitivity for effective and inclusive care. Achieving this goal requires training that recognizes differences and promotes equity, in line with a proposal, developed by Brazilian researchers, called the “Quadrilateral of training for the health area: Teaching, Management, Care and Social Control” [4,10,14,15]. Such a framework aims to prepare professionals to act in line with social rights policies, reducing inequalities and incorporating social and cultural dimensions into healthcare. By valuing social participation and integrated management, the training quadrilateral contributes to more inclusive and equitable practices [16]. Accordingly, it is fully aligned with the intersectional perspective and the social determination of health assumed in this protocol.

Adopting such a perspective is essential when designing curricula that address the care of vulnerable and vulnerabilized populations, such as Black and LGBTQIA+ individuals. Investment in public and universal health systems, with comprehensive, community-based, and territorially grounded primary care, contributes to 1. qualifying health training, preparing workers to meet the specific demands of these groups with technical competence and cultural sensitivity; 2. ensuring comprehensive, equitable care free from ethnic-racial and gender-based violence; and 3. strengthening public and universal health systems as an expression of democratic and decolonial care [16]. The integration of these elements strengthens the capacity of health systems to offer resolutive and humanized responses to social demands, promoting the continuous improvement of the services provided.

The lack of articulation between the quadrilateral of health training and curricula compromises the construction of the profile of workers, having a negative impact on the care and management of services [17]. This compromises the development of the potential needed to welcome and meet the demands of Black and LGBTQIA+ populations.

The absence of these topics in training, especially from an intersectional perspective, further exacerbates the health outcomes of these groups [17]. The identified gap in academic production reinforces the need for studies that contribute to more inclusive and equitable care strategies. The systems of oppression—racism, sexism, machismo, and classicism—act in an intersectional way, aggravating health inequalities globally [6,10,16].

The intentional inclusion of these themes in undergraduate and postgraduate health curricula and their integration into the continuing education agenda is not only desirable, but urgent. This change is essential to strengthen public and universal health systems, making them more equitable and prepared to respond to the challenges of historically vulnerable populations.

A preliminary search of the databases CDSR (Cochrane Database of Systematic Reviews), Epistemonikos, JBI Evidence Synthesis (JBI), MEDLINE (Medical Literature Analysis and Retrieval System Online) and PROSPERO (International Prospective Register of Systematic Reviews) and OSF (Open Science Framework) did not identify any current or ongoing systematic or scoping reviews on the subject. However, three studies in Brazil point to gaps in training: (a) investigated the presence of content on the health of the Black population in medical curricula, pointing to significant gaps and highlighting the need for greater integration of this theme in the training of female workers [18]; (b) analyzed the approach to LGBTQIA+ health in nursing courses, noting the incipient implementation, despite existing guidelines [17]; and (c) reviewed institutional documents from federal universities, identifying initiatives that address the specificities of the health of vulnerable groups, including Black and LGBTQIA+ populations [19].

New studies are urgently needed to consider geographical variations and cultural particularities, providing a more robust overview of health training to welcome and guarantee the vulnerabilities of Black and LGBTQIA+ people. This will enable the development of effective interventions and the formulation of effective public policies in different contexts.

In this sense, unlike these studies [17,18,19], this protocol proposes a review with an international scope, aimed at productions across the globe, and focused on the training of current and future healthcare professionals.

In this way, this review protocol will look at the available evidence as well as the gaps in international and national studies, shedding light on understanding how structural inequalities, based on intersectionalities, cause different experiences of health and training. The intersectional analysis adopted will use foundations from Critical Social Theory by Patricia Hill Collins [2] combining aspects related to territories Rodo Zarate [20], which deal with life experiences in structurally determined geographical, social and symbolic spaces, as well as intersectionality in health, with an emphasis on Black and LGBTQIA+ populations, as proposed by Lisa Bowleg [3].

The aim of this scoping review is to map the scientific evidence and knowledge gaps on how the health of the Black and/or LGBTQIA+ population has been addressed in the training of health workers around the world.

*Review question*

Based on the objective of this review, two guiding questions emerge for the scoping review:

1. What is the current scientific evidence regarding the health of Black and/or LGBTQIA+ populations in the training of current and future healthcare professionals worldwide?
2. What are the existing knowledge gaps in this area?

## 2. Inclusion Criteria

### 2.1. Participants

The present scoping review will include all studies involving health professionals and future health professionals who received training on healthcare for Black and/or LGBTQIA+ populations at the undergraduate, graduate, and other educational levels, such as continuing education and/or permanent education in health [14]. Additionally, institutions and initiatives that incorporate healthcare for these populations into health education will also be considered as participants.

Health education refers to the process of developing the knowledge, skills, and values essential for the professional practice of current and future health professionals. It encompasses initial training in undergraduate programs, as well as stricto and lato sensu graduate programs, in addition to other modalities of continuing education and permanent education in, for, and through health work. For the purposes of this review, educational modalities also include technical and vocational training programs, short-term or free certification courses, and on-the-job training initiatives, particularly relevant in international contexts where the structure of professional development may differ. The educational process under consideration includes pedagogical practices, curricula, teaching methodologies, and public policies, aiming to align the qualifications of health workers and future health workers with the demands of the health system and societal needs. It involves knowledge production, hands-on practice, experimentation, and creativity in healthcare to ensure comprehensiveness, humanization, and quality in the care provided to the population [14,21].

Within this analytical framework, the concept of the quadrilateral of health education encompasses four fundamental elements. The first is teaching, which includes pedagogical and curricular processes. The second is management, which focuses on the organization and administration of health services, as well as the formulation of public policies. The third is healthcare, with an emphasis on comprehensive and equitable care for the population. The fourth is social control, which highlights society’s participation in the planning and oversight of health policies. This approach aims to integrate the training of health workers and future health workers with the demands of the health system and societal needs [14].

In the health sector, professional training extends beyond merely preparing workers and future health professionals for employment. It equips them with essential skills for active listening, where the interaction between the healthcare provider and the user plays a critical role in ensuring high-quality care. In this context, permanent education emerges as a fundamental strategy and tool for the systematic enhancement of these professionals [14].

Permanent Education in Health (PEH) is understood as an educational process that fosters critical reflections on daily practices through the problematization of work processes. Its objective is to transform both the practices of health workers and the organization of services to meet the needs of the population, sectoral management demands, and social control requirements. Additionally, PEH promotes the continuous professional development of health workers by incorporating the latest theoretical, methodological, scientific, and technological advancements [22].

PEH is aligned with different educational approaches. Education in Service involves institutional or political changes in technical training initiatives. Continuing Education focuses on the professional development of health workers within specific contexts. Formal Education of Future Health Workers integrates practical experiences in the workplace with projects linked to the educational sector [22].

### 2.2. Concept

The concept used in this scoping review refers to the health of Black and/or LGBTQIA+ populations.

It is essential to understand that Black individuals belong to racialized groups whose identification is often linked to phenotypic characteristics, such as skin tone, hair type, and facial features. These individuals face structural disadvantages resulting from racism, which include precarious living conditions and limited access to essential social resources, further exacerbated by institutional and interpersonal discrimination [23]. Importantly, racism is not limited to individuals in situations of greater socioeconomic vulnerability; it also affects Black people regardless of their social position. Regarding sexual and gender minorities, this review considers individuals who identify as LGBTQIA+ (lesbian, gay, bisexual, transgender, queer, intersex, asexual, among others). These individuals frequently experience exclusion, discrimination, and violence due to LGBTQIA+ phobia [24].

The health of the Black population is a field dedicated to understanding and addressing the health disparities that affect this group. These inequalities stem from factors such as racism, unfavorable socioeconomic conditions, and unequal access to health services. Issues such as high maternal and infant mortality rates, the prevalence of chronic and infectious diseases, and the impacts of violence exemplify these disparities [4]. Similarly, the specific health needs of LGBTQIA+ populations are often neglected within the health sector, driven by institutional LGBTQIA+ phobia. This field seeks to develop strategies that promote comprehensive health, including the implementation of specific public policies, the qualification and continuous training of health workers for humanized care, and the expansion of access to health services. These efforts aim to address the challenges posed by exclusion and violence within health and social protection systems [20].

Finally, Black individuals and the LGBTQIA+ population experience multiple forms of vulnerability, encompassing subjective, community, and structural dimensions, often exacerbated by negligence and/or omissions by the State. This condition reflects deep-seated inequalities, including racism, sexism, and other forms of oppression, which necessitate inclusive and transformative public policies to promote equity and social justice [25].

### 2.3. Context

Given the nature of this review and its alignment with the JBI methodology [26], no geographical limitations will be applied and all studies addressing health education at a global level will be considered.

The varying conceptions of health systems directly influence the implementation of health policies and, consequently, the structure of health education in different countries, reflecting each nation’s values and priorities. While some countries prioritize universal and public access to healthcare, they still face challenges such as population growth and the increasing influence of the private sector. Conversely, other countries prioritize the medical-industrial complex and the “disease” market, placing economic interests above the broader right to health and equitable access to services, thereby reinforcing a model of a minimal state of rights [27].

These divergences fuel debates on the varying conceptions of rights and universality in the health sector, particularly concerning specific and vulnerable populations, such as Black and LGBTQIA+ communities. In universal health systems, ensuring comprehensive healthcare—both at the individual and collective levels—requires a coordinated network-based organization within territories, beginning with primary care and supported by public administration and service provision. This model reflects “a worldview that values collective well-being, dignity, and human life as fundamental and inalienable principles” [27] (p. 2).

In contrast, selective and non-universal health systems treat health not as a right but as a commodity provided by insurance companies, varying according to the population’s purchasing power. For those without financial resources, access is guaranteed by the state but mediated through the private sector [28,29].

In this regard, it is important to emphasize that, although this study aligns with the strengthening of public, universal, and decolonial health systems, it will consider studies addressing different conceptions and organizational models of health systems. These include public systems with universal access, with or without private complementarity, public social insurance systems, or entirely private systems [16].

## 3. Types of Sources and Study Designs

This scoping review will include descriptive observational study designs, such as case series, individual case reports, and descriptive cross-sectional studies. Analytical observational studies, such as prospective and retrospective cohort studies, case–control studies, and analytical cross-sectional studies, will also be included. Qualitative studies focusing on qualitative data will also be considered, including, but not limited to, designs such as phenomenology, grounded theory, ethnography, qualitative description, action research, and feminist research. Additionally, systematic reviews that meet the inclusion criteria will be considered, depending on the research question. Theses, dissertations, and documentary surveys will also be included in this scoping review.

Experimental and quasi-experimental studies, including randomized controlled trials, non-randomized controlled clinical trials, pre-post studies, and interrupted time-series studies, will be excluded. Furthermore, opinion articles and other non-research texts will not be included.

### 3.1. Methods

The proposed scoping review will be conducted following the JBI methodology for scoping reviews [26] and will be reported in accordance with the Preferred Reporting Items for Systematic Reviews and Meta-Analyses extension for Scoping Reviews (PRISMA-ScR) checklist [30].

Evidence syntheses do not exist in isolation but are part of a broader ‘evidence ecosystem’. Evidence syntheses are recognized as a critical component of care and evidence-based research [26,30]. Currently, JBI endorses eight methodologies for reviews, which make up the evidence ecosystem.

Scoping Reviews, a type of evidence synthesis used to map the key concepts underpinning a field of research, as well as to clarify working definitions and/or the conceptual boundaries of a topic, explore the breadth or extent of the literature. These reviews summarize the evidence, and inform future research, conducted to provide an overview of the evidence or to answer questions about the nature and diversity of evidence/knowledge available. It is important to highlight that there are no assessments of methodological limitations or risks of bias of the included studies. It is also a type of research indicated as a precursor to a systematic review [26,30].

### 3.2. Search Strategy

The search strategy will aim to identify both published and unpublished studies. A three-step search approach will be employed in this review.

First, with the collaboration of a professional librarian, an initial search was conducted in the MEDLINE (Ovid), PubMed, Scopus (Elsevier), and Cumulative Index to Nursing and Allied Health Literature (CINAHL) databases to identify relevant articles on the topic. The text words found in the titles and abstracts of relevant articles, as well as the index terms used to describe them, were analyzed to develop a comprehensive search strategy for MEDLINE (PubMed), Scopus (Elsevier) and CINAHL (EBSCOhost). (see Appendix A).

The search strategy, including all identified keywords and controlled terms, will be adapted to each informational resource included in the review. The reference lists of all primary sources of evidence included in the scoping review will be screened for additional relevant studies. This strategy will undergo peer review by the review team. Studies published in any language will be included, with translations performed when necessary, and no time restrictions will be applied.

The following databases and observatories will be used as informational resources for this review: Scopus (Elsevier), Web of Science, PubMed, PubMed PMC, Embase (Elsevier), MEDLINE, Virtual Health Library (VHL), CINAHL (EBSCOhost), ERIC (EBSCOhost), Cochrane, and Literatura Cinzenta (Brazilian Digital Library of Theses and Dissertations-BDTD). Additionally, ProQuest Dissertations & Theses Global (PQDT-ProQuest Clarivate) and the Networked Digital Library of Theses and Dissertations (NDLTD) will be included.

### 3.3. Study Selection/Evidence Source

After completing the search, all identified studies will be pooled and uploaded to EndNote Web software, where duplicates will be removed [31]. Following a pilot test, two reviewers (DBQ and GSB) will independently screen titles and abstracts to assess their eligibility based on the inclusion criteria established for the review. Potentially relevant sources will be retrieved in full, and their citation details will be imported into Rayyan QCRI (Qatar Computing Research Institute, Doha, Qatar) [32].

The full texts of the selected studies will then be independently evaluated in detail by the same two reviewers (DBQ and GSB) against the inclusion criteria. The reasons for excluding studies that do not meet the inclusion criteria will be recorded and reported in the scoping review. Any disagreements between reviewers at any stage of the selection process will be resolved through discussion, with the involvement of a more experienced third reviewer (BPS) from the scoping review team. The search results and study inclusion process will be fully documented in the final version of the scoping review and presented in a PRISMA flowchart [33].

## 4. Data Extraction

Data will be extracted from the full texts included in the scoping review by two or more independent reviewers using a data extraction tool developed by the review team. The extracted data will include specific details about the participants, concept, context, study methods, and key findings relevant to the review question.

An extraction form was developed for each type of informational resource considered in this review (see Appendix B). The preliminary data extraction tool will be modified and revised as necessary throughout the data extraction process for each included informational resource. Any modifications made will be documented in the scoping review.

Any disagreements between reviewers will be resolved through discussion with one or more additional reviewers. If necessary, the authors of the studies will be contacted to request missing or supplementary data. A pilot test will be conducted using the extraction forms on two or three studies to ensure that all relevant results are accurately extracted.

To enhance the identification of knowledge gaps, we planned to construct a thematic gap matrix mapping the included studies according to (i) geographic region (North America, South America, Europe, Africa, Asia, Oceania, and Multiregional/Global) and (ii) level of education (technical training, undergraduate, postgraduate, and continuing education). This matrix will help visually identify underrepresented areas in the literature and guide future research priorities (Appendix C).

## 5. Analysis and Presentation of Data

The extracted data will be presented in tabular or diagrammatic format, aligned with the objectives of this scoping review. A descriptive summary will accompany the tabulated results, explaining how they relate to the objective and research question of the review.

The first table will present results from primary studies, highlighting the importance of incorporating information from social actors connected in some way to stricto sensu graduate programs. The second table will be constructed using data from websites and gray literature sources, including the Brazilian Digital Library of Theses and Dissertations (BDTD), ProQuest Dissertations & Theses Global (PQDT), and the Networked Digital Library of Theses and Dissertations (NDLTD).

## Data Availability

The original contributions presented in this study are included in the article. Further inquiries can be directed to the corresponding author.

## Public Involvement Statement

There was no public involvement in any aspect of this research.

## Guidelines and Standards Statement

This manuscript was drafted against the JBI methodologies [26] and the PRISMA-ScR [30] for scoping review research.

## Appendix A. Search Strategy

MEDLINE (PubMed), Scopus (ELSEVIER) and CINAHL (EBSCOhost)

Search carried out: 12/11/2024

MEDLINE (PubMed)

**SEARCH**	**QUERY**	**RECORDS RETRIEVED**
#1	(((Black People[MeSH Terms]) OR (“Black People”[Title/Abstract] OR “Black Peoples”[Title/Abstract] OR “People, Black”[Title/Abstract] OR “Black Person”[Title/Abstract] OR “Black Persons”[Title/Abstract] OR “Negroid Race”[Title/Abstract] OR “Negroid Races”[Title/Abstract] OR “Race, Negroid”[Title/Abstract] OR “African Continental Ancestry Group”[Title/Abstract])) OR (“Black Population”[Title/Abstract] OR “Black man”[Title/Abstract] OR “Black race”[Title/Abstract] OR Negroid[Title/Abstract] OR “Negroid race”[Title/Abstract] OR Negroids[Title/Abstract])) OR (((Black or African American[MeSH Terms]) OR (“Black or African American”[Title/Abstract] OR “Black Americans”[Title/Abstract] OR “American, Black”[Title/Abstract] OR “Black American”[Title/Abstract] OR Blacks[Title/Abstract] OR Negroes[Title/Abstract] OR Negro[Title/Abstract] OR “African Americans”[Title/Abstract] OR “African American”[Title/Abstract] OR “American, African”[Title/Abstract] OR “Afro-American”[Title/Abstract] OR “Afro American”[Title/Abstract] OR “Afro-Americans”[Title/Abstract] OR “Afro Americans”[Title/Abstract] OR “African-Americans”[Title/Abstract] OR “African-American”[Title/Abstract])) OR (“American blacks”[Title/Abstract] OR “American Negro”[Title/Abstract] OR “black American”[Title/Abstract] OR “black or African American”[Title/Abstract]))	331,996
#2	((((((Sexual and Gender Minorities[MeSH Terms]) OR (“Sexual and Gender Minorities”[Title/Abstract] OR “LGBT Person”[Title/Abstract] OR “Persons, LGBT”[Title/Abstract] OR “LGBTQ Person”[Title/Abstract] OR “Person, LGBTQ”[Title/Abstract] OR “Persons, LGBTQ”[Title/Abstract] OR “Non-Heterosexual Persons”[Title/Abstract] OR “Non Heterosexual Persons”[Title/Abstract] OR “LBG Persons”[Title/Abstract] OR “Sexual Minorities”[Title/Abstract] OR “Minorities, Sexual”[Title/Abstract] OR “Minority, Sexual”[Title/Abstract] OR “Sexual Minority”[Title/Abstract] OR “Non-Heterosexuals”[Title/Abstract] OR “Non Heterosexuals”[Title/Abstract] OR “Non-Heterosexual”[Title/Abstract] OR “Sexual Dissidents”[Title/Abstract] OR “Sexual Dissident”[Title/Abstract] OR “GLBT Persons”[Title/Abstract] OR “GLBT Person”[Title/Abstract] OR Gays[Title/Abstract] OR Gay[Title/Abstract] OR “Men Who Have Sex With Men”[Title/Abstract] OR Lesbians[Title/Abstract] OR Lesbian[Title/Abstract] OR “Women Who Have Sex With Women”[Title/Abstract] OR Homosexuals[Title/Abstract] OR Homosexual[Title/Abstract])) OR (((“sexual and gender minority”[Title/Abstract]) OR (“LGBTQIA+ people”[Title/Abstract] OR pansexual[Title/Abstract] OR questioning[Title/Abstract] OR “ transgender AND intersex”[Title/Abstract] OR transgender[Title/Abstract] OR “GLBTI+”[Title/Abstract] OR “GLBTQ+”[Title/Abstract] OR “LGBTI+”[Title/Abstract] OR “LGBTIQ+”[Title/Abstract] OR “LGBTIQA+”[Title/Abstract] OR “LGBTIQQ”[Title/Abstract] OR “LGBTQ people”[Title/Abstract] OR “LGBTQ+”[Title/Abstract] OR “LGBTQ2”[Title/Abstract] OR “LGBTQ2S”[Title/Abstract] OR “LGBTQ2SIA+”[Title/Abstract] OR “LGBTQA”[Title/Abstract] OR “LGBTQAI”[Title/Abstract] OR “LGBTQIA”[Title/Abstract] OR “LGBTQIA+”[Title/Abstract] OR “LGBTQIA2S+”[Title/Abstract] OR “LGBTQQ”[Title/Abstract] OR “LGBTQQIA”[Title/Abstract] OR “LGBTTQ+”[Title/Abstract] OR “LGTBQA”[Title/Abstract] OR asexual[Title/Abstract] OR “two-spirit”[Title/Abstract] OR “2-spirit”[Title/Abstract] OR “2 spirit”[Title/Abstract])) OR (LGBTQIAPN+[Title/Abstract]))) OR ((Homosexuality[MeSH Terms]) OR (Homosexuality[Title/Abstract] OR “Ego-Dystonic Homosexuality”[Title/Abstract]))) OR ((Homosexuality, Female[MeSH Terms]) OR (“Homosexuality, Female”[Title/Abstract] OR “Female Homosexuality”[Title/Abstract] OR Lesbianism[Title/Abstract]))) OR ((((Homosexuality, Male[MeSH Terms]) OR (“Homosexuality, Male”[Title/Abstract] OR “Male Homosexuality”[Title/Abstract])) OR (“gender nonbinary”[Title/Abstract] OR “gender non binary”[Title/Abstract] OR “gender non conforming”[Title/Abstract] OR “gender nonconforming”[Title/Abstract] OR “non binary AFAB”[Title/Abstract] OR “non binary gender”[Title/Abstract] OR “non binary gender identity”[Title/Abstract] OR “non binary individuals”[Title/Abstract] OR “non binary people”[Title/Abstract] OR “non conforming gender”[Title/Abstract] OR “nonbinary AFAB”[Title/Abstract] OR “nonbinary gender”[Title/Abstract] OR “nonbinary individuals”[Title/Abstract] OR “nonbinary people”[Title/Abstract])) OR (“gender expansive youth”[Title/Abstract] OR “gender-expansive people”[Title/Abstract] OR “gender-fluid”[Title/Abstract] OR “gender-queer”[Title/Abstract] OR “gender-questioning”[Title/Abstract] OR “genderexpansive”[Title/Abstract] OR genderfluid[Title/Abstract] OR genderqueer[Title/Abstract] OR genderquestioning[Title/Abstract] OR “TGNB people”[Title/Abstract] OR “TNB individuals”[Title/Abstract] OR “TNB people”[Title/Abstract] OR “transgender and gender non-conforming”[Title/Abstract] OR “transgender and gender nonconforming”[Title/Abstract] OR “transgender and nonbinary”[Title/Abstract]))) OR ((“Gender Identity”[MeSH Terms]) OR (“Gender Identity”[Title/Abstract]))	115,367
#3	#1 OR #2	440,838
#4	(((Vulnerable Populations[MeSH Terms]) OR (“Vulnerable Populations”[Title/Abstract] OR “Vulnerable Population”[Title/Abstract] OR “Disadvantaged Populations”[Title/Abstract] OR “Disadvantaged Population”[Title/Abstract] OR “Sensitive Populations”[Title/Abstract] OR “Sensitive Population”[Title/Abstract] OR “Sensitive Population Groups”[Title/Abstract] OR “Sensitive Population Group”[Title/Abstract] OR “Underserved Population”[Title/Abstract] OR “Underserved Populations”[Title/Abstract])) OR (“vulnerable minorities”[Title/Abstract] OR “vulnerable minority”[Title/Abstract] OR “vulnerable minority population”[Title/Abstract] OR “vulnerable people”[Title/Abstract] OR “vulnerable person”[Title/Abstract] OR “vulnerable persons”[Title/Abstract])) OR (((Social Vulnerability[MeSH Terms]) OR (“Social Vulnerability”[Title/Abstract] OR “Social Vulnerabilities”[Title/Abstract])) OR (Vulnerability[Title/Abstract] OR Vulnerable[Title/Abstract]))	217,456
#5	((Health Inequities[MeSH Terms]) OR (“Health Inequities”[Title/Abstract] OR “Health Inequity”[Title/Abstract] OR “Health Inequalities”[Title/Abstract] OR “Health Inequality”[Title/Abstract] OR “Health Disparities”[Title/Abstract] OR “Health Disparity”[Title/Abstract])) OR (“disparity in health”[Title/Abstract] OR “health status disparities”[Title/Abstract] OR “health status disparity”[Title/Abstract] OR “inequality in health”[Title/Abstract] OR “inequity in health”[Title/Abstract] OR “socioeconomic disparities in health”[Title/Abstract])	74,596
#6	(health[Title/Abstract]) AND (Inequities[Title/Abstract] OR Inequity[Title/Abstract] OR Inequalities[Title/Abstract] OR Inequality[Title/Abstract] OR Disparities[Title/Abstract] OR Disparity[Title/Abstract])	107,580
#7	#5 OR #6	129,386
#8	#4 AND #7	11,061
#9	((((((((Teaching[MeSH Terms]) OR (Teaching[Title/Abstract] OR “Training Techniques”[Title/Abstract] OR “Training Technique”[Title/Abstract] OR “Training Technics”[Title/Abstract] OR “Training Technic”[Title/Abstract] OR Pedagogy[Title/Abstract] OR Pedagogies[Title/Abstract] OR “Teaching Methods”[Title/Abstract] OR “Teaching Method”[Title/Abstract] OR “Academic Training”[Title/Abstract] OR “Training Activities”[Title/Abstract] OR “Training Activity”[Title/Abstract] OR “Educational Techniques”[Title/Abstract] OR “Educational Technique”[Title/Abstract] OR “Educational Technics”[Title/Abstract] OR “Educational Technic”[Title/Abstract])) OR (((Curriculum[MeSH Terms]) OR (Curriculum[Title/Abstract] OR Curricula[Title/Abstract] OR “Short-Term Courses”[Title/Abstract] OR “Short Term Courses”[Title/Abstract])) OR (“competency-based education”[Title/Abstract] OR “integrated curriculum”[Title/Abstract]))) OR ((Education, Continuing[MeSH Terms]) OR (“Education, Continuing”[Title/Abstract] OR “Continuous Learning”[Title/Abstract] OR “Lifelong Learning”[Title/Abstract] OR “Life-Long Learning”[Title/Abstract] OR “Life Long Learning” “Continuing Education”[Title/Abstract]))) OR ((Education, Professional[MeSH Terms]) OR (“Education, Professional”[Title/Abstract] OR “Professional Education”[Title/Abstract]))) OR (((Interprofessional Education[MeSH Terms]) OR (“Interprofessional Education”[Title/Abstract] OR “Education, Interprofessional”[Title/Abstract])) OR (“inter-professional education”[Title/Abstract] OR “cross training”[Title/Abstract] OR multi-skilling[Title/Abstract] OR multiskilling[Title/Abstract]))) OR (“Professional Training”[Title/Abstract])) OR ((Schools[MeSH Terms]) OR (Schools[Title/Abstract] OR School[Title/Abstract] OR “Secondary School”[Title/Abstract] OR “Secondary Schools”[Title/Abstract]))) OR ((Health Personnel[MeSH Terms]) OR (“Health Personnel”[Title/Abstract] OR “Healthcare Workers”[Title/Abstract] OR “Healthcare Worker”[Title/Abstract] OR “Health Care Providers”[Title/Abstract] OR “Health Care Provider”[Title/Abstract] OR “Healthcare Providers”[Title/Abstract] OR “Healthcare Provider”[Title/Abstract] OR “Health Care Professionals”[Title/Abstract] OR “Health Care Professional”[Title/Abstract]))	1,548,041
#10	#3 AND #8 AND #9	353

Scopus (ELSEVIER)

**SEARCH**	**QUERY**	**RECORDS RETRIEVED**
#1	(TITLE-ABS-KEY (“Black People” OR “Black Peoples” OR “People, Black” OR “Black Person” OR “Black Persons” OR “Negroid Race” OR “Negroid Races” OR “Race, Negroid” OR “African Continental Ancestry Group”) OR TITLE-ABS-KEY (“Black Population” OR “Black man” OR “Black race” OR negroid OR “Negroid race” OR negroids) OR TITLE-ABS-KEY (“Black or African American” OR “Black Americans” OR “American, Black” OR “Black American” OR blacks OR negroes OR negro OR “African Americans” OR “African American” OR “American, African” OR “Afro-American” OR “Afro American” OR “Afro-Americans” OR “Afro Americans” OR “African-Americans” OR “African-American”) OR TITLE-ABS-KEY (“American blacks” OR “American Negro” OR “black American” OR “black or African American”)	828,909
#2	(TITLE-ABS-KEY (“Sexual and Gender Minorities” OR “LGBT Person” OR “Persons, LGBT” OR “LGBTQ Person” OR “Person, LGBTQ” OR “Persons, LGBTQ” OR “Non-Heterosexual Persons” OR “Non Heterosexual Persons” OR “LBG Persons” OR “Sexual Minorities” OR “Minorities, Sexual” OR “Minority, Sexual” OR “Sexual Minority” OR “Non-Heterosexuals” OR “Non Heterosexuals” OR “Non-Heterosexual” OR “Sexual Dissidents” OR “Sexual Dissident” OR “GLBT Persons” OR “GLBT Person” OR gays OR gay OR “Men Who Have Sex With Men” OR lesbians OR lesbian OR “Women Who Have Sex With Women” OR homosexuals OR homosexual) OR TITLE-ABS-KEY (“sexual and gender minority”) OR TITLE-ABS-KEY (“LGBTQIA+ people” OR pansexual OR questioning OR “ transgender and intersex” OR transgender OR “GLBTI+” OR “GLBTQ+” OR “LGBTI+” OR “LGBTIQ+” OR “LGBTIQA+” OR “LGBTIQQ” OR “LGBTQ people” OR “LGBTQ+” OR “LGBTQ2” OR “LGBTQ2S” OR “LGBTQ2SIA+” OR “LGBTQA” OR “LGBTQAI” OR “LGBTQIA” OR “LGBTQIA+” OR “LGBTQIA2S+” OR “LGBTQQ” OR “LGBTQQIA” OR “LGBTTQ+” OR “LGTBQA” OR asexual OR “two-spirit” OR “2-spirit” OR “2 spirit”) OR TITLE-ABS-KEY (lgbtqiapn+) OR TITLE-ABS-KEY (homosexuality OR “Ego-Dystonic Homosexuality”) OR TITLE-ABS-KEY (“Homosexuality, Female” OR “Female Homosexuality” OR lesbianism) OR TITLE-ABS-KEY (“Homosexuality, Male” OR “Male Homosexuality”) OR TITLE-ABS-KEY (“gender nonbinary” OR “gender non binary” OR “gender non conforming” OR “gender nonconforming” OR “non binary AFAB” OR “non binary gender” OR “non binary gender identity” OR “non binary individuals” OR “non binary people” OR “non conforming gender” OR “nonbinary AFAB” OR “nonbinary gender” OR “nonbinary individuals” OR “nonbinary people”) OR TITLE-ABS-KEY (“gender expansive youth” OR “gender-expansive people” OR “gender-fluid” OR “gender-queer” OR “gender-questioning” OR “genderexpansive” OR genderfluid OR genderqueer OR genderquestioning OR “TGNB people” OR “TNB individuals” OR “TNB people” OR “transgender and gender non-conforming” OR “transgender and gender nonconforming” OR “transgender and nonbinary”) OR TITLE-ABS-KEY (“Gender Identity”)	230,709
#3	#1 OR #2	1,049,375
#4	(TITLE-ABS-KEY (“Vulnerable Populations” OR “Vulnerable Population” OR “Disadvantaged Populations” OR “Disadvantaged Population” OR “Sensitive Populations” OR “Sensitive Population” OR “Sensitive Population Groups” OR “Sensitive Population Group” OR “Underserved Population” OR “Underserved Populations”) OR TITLE-ABS-KEY (“vulnerable minorities” OR “vulnerable minority” OR “vulnerable minority population” OR “vulnerable people” OR “vulnerable person” OR “vulnerable persons”) OR TITLE-ABS-KEY (“Social Vulnerability” OR “Social Vulnerabilities”) OR TITLE-ABS-KEY (vulnerability OR vulnerable)	595,443
#5	(TITLE-ABS-KEY (“Health Inequities” OR “Health Inequity” OR “Health Inequalities” OR “Health Inequality” OR “Health Disparities” OR “Health Disparity”) OR TITLE-ABS-KEY (“disparity in health” OR “health status disparities” OR “health status disparity” OR “inequality in health” OR “inequity in health” OR “socioeconomic disparities in health”)	86,736
#6	(TITLE-ABS-KEY (health) AND TITLE-ABS-KEY (inequities OR inequity OR inequalities OR inequality OR disparities OR disparity)	193,540
#7	#5 OR #6	193,540
#8	#4 AND #7	17,107
#9	(TITLE-ABS-KEY (teaching OR “Training Techniques” OR “Training Technique” OR “Training Technics” OR “Training Technic” OR pedagogy OR pedagogies OR “Teaching Methods” OR “Teaching Method” OR “Academic Training” OR “Training Activities” OR “Training Activity” OR “Educational Techniques” OR “Educational Technique” OR “Educational Technics” OR “Educational Technic”) OR TITLE-ABS-KEY (curriculum OR curricula OR “Short-Term Courses” OR “Short Term Courses”) OR TITLE-ABS-KEY (“competency-based education” OR “integrated curriculum”) OR TITLE-ABS-KEY (“Education, Continuing” OR “Continuous Learning” OR “Lifelong Learning” OR “Life-Long Learning” OR “Life Long Learning” “Continuing Education”) OR TITLE-ABS-KEY (“Education, Professional” OR “Professional Education”) OR TITLE-ABS-KEY (“Interprofessional Education” OR “Education, Interprofessional”) OR TITLE-ABS-KEY (“inter-professional education” OR “cross training” OR multi-skilling OR multiskilling) OR TITLE-ABS-KEY (“Professional Training”) OR TITLE-ABS-KEY (schools OR school OR “Secondary School” OR “Secondary Schools”) OR TITLE-ABS-KEY (“Health Personnel” OR “Healthcare Workers” OR “Healthcare Worker” OR “Health Care Providers” OR “Health Care Provider” OR “Healthcare Providers” OR “Healthcare Provider” OR “Health Care Professionals” OR “Health Care Professional”)	2,805,281
#10	#3 AND #8 AND #8	444

CINAHL (EBSCOhost)

**SEARCH**	**QUERY**	**RECORDS RETRIEVED**
#1	(MM “Black Persons+”) OR TI (“Black People” OR “Black Peoples” OR “People, Black” OR “Black Person” OR “Black Persons” OR “Negroid Race” OR “Negroid Races” OR “Race, Negroid” OR “African Continental Ancestry Group”) OR AB (“Black People” OR “Black Peoples” OR “People, Black” OR “Black Person” OR “Black Persons” OR “Negroid Race” OR “Negroid Races” OR “Race, Negroid” OR “African Continental Ancestry Group”) OR TI (“Black Population” OR “Black man” OR “Black race” OR Negroid OR “Negroid race” OR Negroids) OR AB (“Black Population” OR “Black man” OR “Black race” OR Negroid OR “Negroid race” OR Negroids) OR TI (“Black or African American” OR “Black Americans” OR “American, Black” OR “Black American” OR Blacks OR Negroes OR Negro OR “African Americans” OR “African American” OR “American, African” OR “Afro-American” OR “Afro American” OR “Afro-Americans” OR “Afro Americans” OR “African-Americans” OR “African-American”) OR AB (“Black or African American” OR “Black Americans” OR “American, Black” OR “Black American” OR Blacks OR Negroes OR Negro OR “African Americans” OR “African American” OR “American, African” OR “Afro-American” OR “Afro American” OR “Afro-Americans” OR “Afro Americans” OR “African-Americans” OR “African-American”) OR TI (“American blacks” OR “American Negro” OR “black American” OR “black or African American”) OR AB (“American blacks” OR “American Negro” OR “black American” OR “black or African American”)	87,873
#2	(MM “Sexual and Gender Minorities+”) OR TI (“Sexual and Gender Minorities” OR “LGBT Person” OR “Persons, LGBT” OR “LGBTQ Person” OR “Person, LGBTQ” OR “Persons, LGBTQ” OR “Non-Heterosexual Persons” OR “Non Heterosexual Persons” OR “LBG Persons” OR “Sexual Minorities” OR “Minorities, Sexual” OR “Minority, Sexual” OR “Sexual Minority” OR “Non-Heterosexuals” OR “Non Heterosexuals” OR “Non-Heterosexual” OR “Sexual Dissidents” OR “Sexual Dissident” OR “GLBT Persons” OR “GLBT Person” OR Gays OR Gay OR “Men Who Have Sex With Men” OR Lesbians OR Lesbian OR “Women Who Have Sex With Women” OR Homosexuals OR Homosexual) OR AB (“Sexual and Gender Minorities” OR “LGBT Person” OR “Persons, LGBT” OR “LGBTQ Person” OR “Person, LGBTQ” OR “Persons, LGBTQ” OR “Non-Heterosexual Persons” OR “Non Heterosexual Persons” OR “LBG Persons” OR “Sexual Minorities” OR “Minorities, Sexual” OR “Minority, Sexual” OR “Sexual Minority” OR “Non-Heterosexuals” OR “Non Heterosexuals” OR “Non-Heterosexual” OR “Sexual Dissidents” OR “Sexual Dissident” OR “GLBT Persons” OR “GLBT Person” OR Gays OR Gay OR “Men Who Have Sex With Men” OR Lesbians OR Lesbian OR “Women Who Have Sex With Women” OR Homosexuals OR Homosexual) OR TI (“sexual and gender minority”) OR AB (“sexual and gender minority”) OR TI (“LGBTQIA+ people” OR pansexual OR questioning OR “ transgender and intersex” OR transgender OR “GLBTI+” OR “GLBTQ+” OR “LGBTI+” OR “LGBTIQ+” OR “LGBTIQA+” OR “LGBTIQQ” OR “LGBTQ people” OR “LGBTQ+” OR “LGBTQ2” OR “LGBTQ2S” OR “LGBTQ2SIA+” OR “LGBTQA” OR “LGBTQAI” OR “LGBTQIA” OR “LGBTQIA+” OR “LGBTQIA2S+” OR “LGBTQQ” OR “LGBTQQIA” OR “LGBTTQ+” OR “LGTBQA” OR asexual OR “two-spirit” OR “2-spirit” OR “2 spirit”) OR AB (“LGBTQIA+ people” OR pansexual OR questioning OR “ transgender and intersex” OR transgender OR “GLBTI+” OR “GLBTQ+” OR “LGBTI+” OR “LGBTIQ+” OR “LGBTIQA+” OR “LGBTIQQ” OR “LGBTQ people” OR “LGBTQ+” OR “LGBTQ2” OR “LGBTQ2S” OR “LGBTQ2SIA+” OR “LGBTQA” OR “LGBTQAI” OR “LGBTQIA” OR “LGBTQIA+” OR “LGBTQIA2S+” OR “LGBTQQ” OR “LGBTQQIA” OR “LGBTTQ+” OR “LGTBQA” OR asexual OR “two-spirit” OR “2-spirit” OR “2 spirit”) OR TI LGBTQIAPN+ OR AB LGBTQIAPN+ OR (MM “Homosexuality”) OR TI (Homosexuality OR “Ego-Dystonic Homosexuality”) OR AB (Homosexuality OR “Ego-Dystonic Homosexuality”) OR (MM “Lesbians”) OR TI (Homosexuality, Female” OR “Female Homosexuality” OR Lesbianism) OR AB (Homosexuality, Female” OR “Female Homosexuality” OR Lesbianism) OR TI (“Homosexuality, Male” OR “Male Homosexuality”) OR AB (“Homosexuality, Male” OR “Male Homosexuality”) OR TI (“gender nonbinary” OR “gender non binary” OR “gender non conforming” OR “gender nonconforming” OR “non binary AFAB” OR “non binary gender” OR “non binary gender identity” OR “non binary individuals” OR “non binary people” OR “non conforming gender” OR “nonbinary AFAB” OR “nonbinary gender” OR “nonbinary individuals” OR “nonbinary people”) OR AB (“gender nonbinary” OR “gender non binary” OR “gender non conforming” OR “gender nonconforming” OR “non binary AFAB” OR “non binary gender” OR “non binary gender identity” OR “non binary individuals” OR “non binary people” OR “non conforming gender” OR “nonbinary AFAB” OR “nonbinary gender” OR “nonbinary individuals” OR “nonbinary people”) OR TI (“gender expansive youth” OR “gender-expansive people” OR “gender-fluid” OR “gender-queer” OR “gender-questioning” OR “genderexpansive” OR genderfluid OR genderqueer OR genderquestioning OR “TGNB people” OR “TNB individuals” OR “TNB people” OR “transgender and gender non-conforming” OR “transgender and gender nonconforming” OR “transgender and nonbinary”) OR AB (“gender expansive youth” OR “gender-expansive people” OR “gender-fluid” OR “gender-queer” OR “gender-questioning” OR “genderexpansive” OR genderfluid OR genderqueer OR genderquestioning OR “TGNB people” OR “TNB individuals” OR “TNB people” OR “transgender and gender non-conforming” OR “transgender and gender nonconforming” OR “transgender and nonbinary”) OR (MM “Gender Identity+”) OR TI “Gender Identity” OR AB “Gender Identity”	42,503
#3	#1 OR #2	127,695
#4	(MM “Special Populations”) OR TI (“Vulnerable Populations” OR “Vulnerable Population” OR “Disadvantaged Populations” OR “Disadvantaged Population” OR “Sensitive Populations” OR “Sensitive Population” OR “Sensitive Population Groups” OR “Sensitive Population Group” OR “Underserved Population” OR “Underserved Populations”) OR AB (“Vulnerable Populations” OR “Vulnerable Population” OR “Disadvantaged Populations” OR “Disadvantaged Population” OR “Sensitive Populations” OR “Sensitive Population” OR “Sensitive Population Groups” OR “Sensitive Population Group” OR “Underserved Population” OR “Underserved Populations”) OR TI (“vulnerable minorities” OR “vulnerable minority” OR “vulnerable minority population” OR “vulnerable people” OR “vulnerable person” OR “vulnerable persons”) OR AB (“vulnerable minorities” OR “vulnerable minority” OR “vulnerable minority population” OR “vulnerable people” OR “vulnerable person” OR “vulnerable persons”) OR TI (“Social Vulnerability” OR “Social Vulnerabilities”) OR AB (“Social Vulnerability” OR “Social Vulnerabilities”) OR TI (Vulnerability OR Vulnerable) OR AB (Vulnerability OR Vulnerable)	71,714
#5	(MM “Health Inequities”) OR TI (“Health Inequities” OR “Health Inequity” OR “Health Inequalities” OR “Health Inequality” OR “Health Disparities” OR “Health Disparity”) OR AB (“Health Inequities” OR “Health Inequity” OR “Health Inequalities” OR “Health Inequality” OR “Health Disparities” OR “Health Disparity”) OR TI (“disparity in health” OR “health status disparities” OR “health status disparity” OR “inequality in health” OR “inequity in health” OR “socioeconomic disparities in health”) OR AB (“disparity in health” OR “health status disparities” OR “health status disparity” OR “inequality in health” OR “inequity in health” OR “socioeconomic disparities in health”)	19,807
#6	TI health OR AB health AND TI (Inequities OR Inequity OR Inequalities OR Inequality OR Disparities OR Disparity) OR AB (Inequities OR Inequity OR Inequalities OR Inequality OR Disparities OR Disparity)	48,713
#7	#5 OR #6	49,281
#8	#4 AND #7	4,032
#9	(MM “Teaching+”) OR TI (Teaching OR “Training Techniques” OR “Training Technique” OR “Training Technics” OR “Training Technic” OR Pedagogy OR Pedagogies OR “Teaching Methods” OR “Teaching Method” OR “Academic Training” OR “Training Activities” OR “Training Activity” OR “Educational Techniques” OR “Educational Technique” OR “Educational Technics” OR “Educational Technic”) OR AB (Teaching OR “Training Techniques” OR “Training Technique” OR “Training Technics” OR “Training Technic” OR Pedagogy OR Pedagogies OR “Teaching Methods” OR “Teaching Method” OR “Academic Training” OR “Training Activities” OR “Training Activity” OR “Educational Techniques” OR “Educational Technique” OR “Educational Technics” OR “Educational Technic”) OR (MM “Curriculum+”) OR TI (Curriculum OR Curricula OR “Short-Term Courses” OR “Short Term Courses”) OR AB (Curriculum OR Curricula OR “Short-Term Courses” OR “Short Term Courses”) OR TI (“competency-based education” OR “integrated curriculum”) OR AB (“competency-based education” OR “integrated curriculum”) OR (MM “Education, Continuing+”) OR TI (“Education, Continuing” OR “Continuous Learning” OR “Lifelong Learning” OR “Life-Long Learning” OR “Life Long Learning” “Continuing Education”) OR AB (“Education, Continuing” OR “Continuous Learning” OR “Lifelong Learning” OR “Life-Long Learning” OR “Life Long Learning” “Continuing Education”) OR TI (“Education, Professional” OR “Professional Education”) OR AB (“Education, Professional” OR “Professional Education”) OR (MM “Education, Interdisciplinary”) OR TI (“Interprofessional Education” OR “Education, Interprofessional”) OR AB (“Interprofessional Education” OR “Education, Interprofessional”) OR TI (“inter-professional education” OR “cross training” OR multi-skilling OR multiskilling) OR AB (“inter-professional education” OR “cross training” OR multi-skilling OR multiskilling) OR TI “Professional Training” OR AB “Professional Training” OR (MM “Schools+”) OR TI (Schools OR School OR “Secondary School” OR “Secondary Schools”) OR AB (Schools OR School OR “Secondary School” OR “Secondary Schools”) OR (MM “Health Personnel+”) OR TI (“Health Personnel” OR “Healthcare Workers” OR “Healthcare Worker” OR “Health Care Providers” OR “Health Care Provider” OR “Healthcare Providers” OR “Healthcare Provider” OR “Health Care Professionals” OR “Health Care Professional”) OR AB (“Health Personnel” OR “Healthcare Workers” OR “Healthcare Worker” OR “Health Care Providers” OR “Health Care Provider” OR “Healthcare Providers” OR “Healthcare Provider” OR “Health Care Professionals” OR “Health Care Professional”)	
#10	#3 AND #8 AND #9	146

## Appendix B. Data Extraction Instrument

### Appendix B.1. For Primary Studies

Workers and future health workers or institutions and initiatives, Appendix B.1 and Appendix B.2, respectively; see participants.

**Author(s)**	
**Year of Publication**	
Study Design (Type)	
Objective(s)	
Context	Region/State
	Developed Location (University, Health Service, Community, etc.)
Participants (Health Workers and Future Health Professionals)	Occupation/Field of Expertise
	Race/Ethnicity
	Sex/Gender Identity
	Age (Mean/Median)
	Educational Level
Concept	Topics Covered: HBP or Health of the LGBTQIA+ Population
	Methodological Approach
	Course, Subject, or Class (Curricular or Standalone Initiative)
	Objective of the Educational Approach
	Results achieved
	Limitations and Recommendations

### Appendix B.2. For Theses and Dissertations

**Author(s)**	
**Year of Publication**	
Study Design (Type)	
Objective(s)	
Context	Region/State
	Setting (University, health service, community, etc.)
Participants (Institutions and Initiatives	Type: University, Research Centers, Health Services: health centers, hospitals, outpatient clinics.
	Sector: Public, private, philanthropic, or mixed institution
	Field: Health, Education, Research, Assistance, Management.
Concept	Topics Covered: HBP or Health of the LGBTQIA+ Population HBP or Health of the LGBTI+ population
	Methodological Approach
	Course, Subject, or Class (Curricular or Standalone Initiative)
	Objective of the Educational Approach
	Results achieved
	Limitations and Recommendations

### Appendix B.3. For Systematic and Scoping Reviews

**Author(s)**	
**Year of Publication**	
Objective(s) of the Review	
Eligibility Criteria for the Population (as defined by the authors for population selection).	
Eligibility Criteria for Study Selection (as defined by the authors)	
Eligibility Criteria for Publication (language, publication period/year, location)	
Number of studies included	
Concept	Topics Covered: HBP or Health of the LGBTQIA+ Population HBP or Health of the LGBTI+ population
	Methodological Approach
	Course, Subject, or Class (curricular or standalone initiative)
	Objective of the Educational Approach
Results achieved	
Limitations and Recommendations	

## Appendix C. Thematic Gap Matrix by Region and Level of Education

**Region/Education Level**	**Technical Training**	**Undergraduate Education**	**Postgraduate Education**	**Continuing Education/In-Service Training**
North America				
South America				
Europe				
Africa				
Asia				
Oceania				
Multiregional/Global				
